# MiR542-3p Regulates the Epithelial-Mesenchymal Transition by Directly Targeting BMP7 in NRK52e

**DOI:** 10.3390/ijms161126075

**Published:** 2015-11-24

**Authors:** Zhicheng Liu, Yuru Zhou, Yue Yuan, Fang Nie, Rui Peng, Qianyin Li, Zhongshi Lyu, Zhaomin Mao, Liyuan Huang, Li Zhou, Yiman Li, Jing Hao, Dongsheng Ni, Qianni Jin, Yaoshui Long, Pan Ju, Wen Yu, Jianing Liu, Yanxia Hu, Qin Zhou

**Affiliations:** 1 The Division of Molecular Nephrology and the Creative Training Center for Undergraduates, The M.O.E. Key Laboratory of Laboratory Medical Diagnostics, the College of Laboratory Medicine, Chongqing Medical University, Chongqing 400016, China; liuzhicheng323@163.com (Z.L.); yyokyy1126@hotmail.com (Y.Y.); nifang168@163.com (F.N.); pengrui911@foxmail.com (R.P.); lqianyincqmu@163.com (Q.L.); Zhongshilyu@163.com (Z.L.); mao1204086118@163.com (Z.M.); lyhuang0603@sina.com (L.H.); shmily900519@foxmail.com (L.Z.); 18715854036@sina.cn (Y.L.); lanyxiu@163.com (J.H.); cqmunds@163.com (D.N.); qiannijincqmu@sina.com (Q.J.); lys960110@sina.com (Y.L.); 18883936591@163.com (P.J.); yuwen0717@sina.com (W.Y.); keithljn@sina.cn (J.L.); hyx_zuichu@outlook.com (Y.H.); 2 The Seventh Class of 2012 year entry, the Third Clinical College, Chongqing Medical University, Chongqing 400016, China; zhouyuru93@163.com

**Keywords:** kidney fibrosis, miR542-3p, bone morphogenetic protein 7 (BMP7), Epithelial-Eesenchymal Transition (EMT), transforming growth factor beta 1 (TGFβ1)

## Abstract

Accumulating evidence demonstrated that miRNAs are highly involved in kidney fibrosis and Epithelial-Eesenchymal Transition (EMT), however, the mechanisms of miRNAs in kidney fibrosis are poorly understood. In this work, we identified that miR542-3p could promote EMT through down-regulating bone morphogenetic protein 7 (BMP7) expression by targeting BMP7 3′UTR. Firstly, real-time PCR results showed that miR542-3p was significantly up-regulated in kidney fibrosis *in vitro* and *in vivo*. Moreover, Western blot results demonstrated that miR542-3p may promote EMT in the NRK52e cell line. In addition, we confirmed that BMP7, which played a crucial role in anti-kidney fibrosis and suppressed the progression of EMT, was a target of miR542-3p through Dual-Luciferase reporter assay, as did Western blot analysis. The effects of miR542-3p on regulating EMT could also be suppressed by transiently overexpressing BMP7 in NRK52e cells. Taken together, miR542-3p may be a critical mediator of the induction of EMT via directly targeting BMP7.

## 1. Introduction

Kidney fibrosis, characterized by the abnormal accumulation of the interstitial extracellular matrix (ECM) and myofibroblast, kept a delicate balance between the fibrosis factor and the anti-fibrosis factor [1,2,3]. Stimuli in chronic kidney diseases, such as Inflammation-Oxidative stress [4,5,6], Transforming Growth Factor signaling pathway activation, extracellular matrix stabilizers [7], and the change of miRNAs expression levels, were the common driving force of the kidney fibrosis and Epithelial-to-Mesenchymal (EMT).

BMP7 protein, a crucial factor in developmental abnormalities and diseases, had a wide range of functions [8,9,10,11,12,13,14,15]. The absence of BMP7 was a vital cause of perinatal mortality with kidney stunting and malformations. Previous studies showed that nephron progenitor cells were defected and glomerulus formation ceased in the absence of BMP7 induced conditions [14,16]. Accumulating evidence also indicated that BMP7 had a beneficial effect on kidney function, and also played an important role in inhibiting the progression of renal fibrosis in the mouse model of unilateral urethral obstruction [17,18]. Additionally, the BMP7 protein levels would dramatically decrease while suffering from kidney diseases, however, the expression would restore after the recovery of tubular and glomerular damage [17].

MicroRNAs, a family of small and noncoding endogenous RNAs, played a vital role in diverse biological and pathological processes, including cell proliferation, differentiation, apoptosis, and particularly in organ fibrosis through regulating target gene expression at the post-transcription [19,20,21,22,23,24,25,26]. Previous findings explained that the abnormal or ectopic expression of microRNAs could affect several aspects of the pathogenesis of renal fibrosis by regulating the expression of target genes, such as miR21 [27,28,29,30] and miR192 [31,32]. Additionally, researchers have stated that miR542-3p participates in several processes of physiology and pathology [19,22,23,26,33,34]. For instance, tumor progression by targeting surviving mRNA [21,26] and down-regulating Integrin-Linked Kinase (ILK) [35]; up-regulating p53 by weakening the stability of MDM2 [22]; regulating steoblast cell proliferation and differentiation by decreasing BMP7. However, limited information about the precise role of miR542-3p in Epithelial-to-Mesenchymal Transition (EMT) was available.

In order to investigate whether miR542-3p have participated in the kidney fibrosis and the EMT progression, the unilateral ureteral obstruction (UUO) mice and TGFβ1-induced NRK52e cell line were used as models of fibrosis *in vitro* and *in vivo* respectively. We first demonstrated that miR542-3p was up-regulated while fibrosis *in vivo* and its expression also could be activated by TGFβ1 *in vitro*. Subsequently, we performed a series of experiments to determine whether miR542-3p could directly interact with BMP7 3′UTR and promote the EMT progression through regulating BMP7 protein expression levels. Moreover, overexpression of BMP7 could attenuate the miR542-3p function of promoting EMT progression in the NRK52e cell. As a result, we hypothesized that miR542-3p played a crucial role in the TGFβ1-induced kidney fibrosis through reducing the expression of BMP7, and that miR542-3p may be a potential therapeutic target of anti-EMT in kidney fibrosis.

## 2. Results

### 2.1. MiR542-3p Expression Was Up-Regulated in Fibrotic Kidney

**Figure 1:**
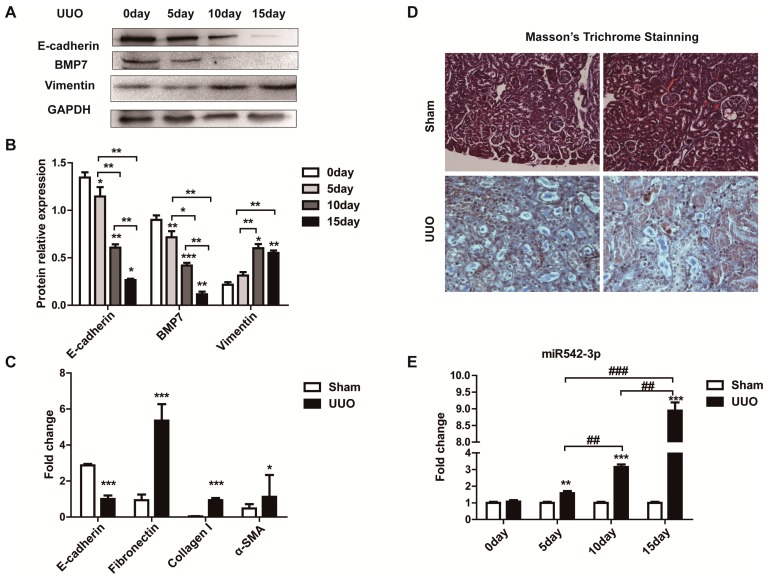
MiR542-3p up-regulation in the unilateral ureteral obstruction (UUO) mice model. (**A**) Compared with control groups, the E-cadherin and BMP7 proteins were reduced in the UUO kidney; (**B**) Histogram showed the gray scale quantitative analysis for Western blot, * *p* < 0.05, ** *p* < 0.01, *** *p* < 0.001; (**C**) The change of expression levels of Epithelial-Eesenchymal Transition (EMT) and fibrotic marker’s mRNA levels, * *p* < 0.05, *** *p* < 0.001, mRNAs expression were normalized to 18sRNA; (**D**) Masson’s Trichrome staining; (**E**) The miR542-3p expression levels was elevated in kidney of UUO mice model, ** *p* < 0.01, *** *p* < 0.001, **^##^** *p* < 0.01, **^###^** *p* < 0.001 the miR542-3p expression was normalized to U6. All data are presented as means ± SD from three independent experiments.

To explore whether miR542-3p was involved in the kidney fibrosis and Epithelial-Mesenchymal Transition (EMT), we firstly constructed a kidney fibrosis model through unilateral ureteral obstruction (UUO). According to Western blot results (Figure 1A,B) and Masson’s Trichrome (Figure 1D) staining, we confirmed the fibrosis kidney. We then investigated the fibrotic and EMT markers’ expression levels in this model (UUO), the results of which indicated that a substantial amount of Collagen I, α-SMA, and Fibroncetin (Figure 1C) were accumulated while the E-cadherin (Figure 1A,C) was decreased. In order to investigate the expression pattern of miR542-3p in a kidney fibrosis mice model (Figure 1E), we tested its expression level on different days post-UUO treatment by quantitative real-time PCR with a special primer. Compared to the normal kidney, the expression of miR542-3p was significantly increased in the kidneys of the UUO mice model (Figure 1E). In general, we suspected that up-regulation of miR542-3p may be closely related to the kidney fibrosis and EMT progression.

### 2.2. Identification of TGFβ1 as a Positive Regulator of MiR542-3p Expression

**Figure 2:**
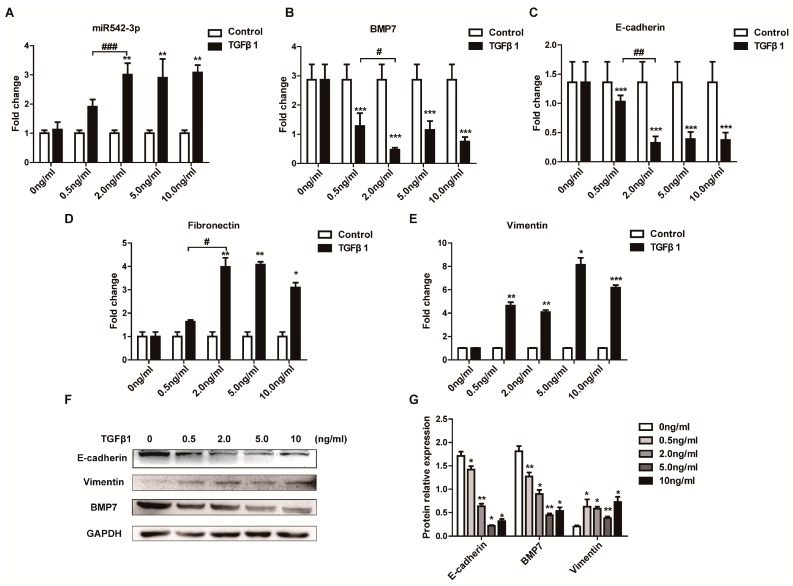
MiR542-3p expression regulated by TGFβ1. (**A**) miR542-3p expression elevated in the NRK52e cell line induced by different TGFβ1 concentration, the peaking concentration is 2 ng/mL; (**B**–**E**) The BMP7 and E-cadherin expressions were opposite to the expression of miR542-3p in the NRK52e cell line induced by different TGFβ1 concentrations. However the expression tendency of Fibronectin and Vimentin are just like that of miR542-3p in the NRK52e cell line induced by different TGFβ1 concentrations; (**F**) The expression level of BMP7, the epithelial marker, and the mesenchymal marker in NRK52e cells treated with different TGFβ1 concentrations; (**G**) Histogram shows the gray scale quantitative analysis for Western blot. All data are displayed as means ± SD from three independent experiments, * *p* < 0.05, ** *p* < 0.01, *** *p* < 0.001 *versus* NRK52e cells without being pre-treated by TGFβ1; **^#^** *p* < 0.05, **^##^** *p* < 0.01, **^###^** *p* < 0.001 *versus* NRK52e cells pre-treated by 0.5 ng/mL TGFβ1, and miR542-3p and mRNAs were normalized to U6 and 18sRNA respectively.

**Figure 3:**
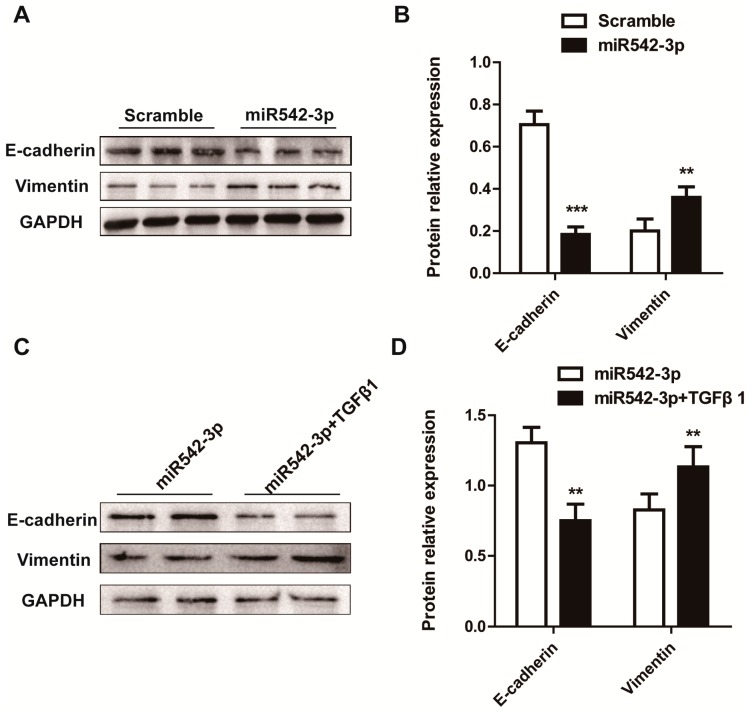
MiR542-3p promotes Epithelial-Mesenchymal Transition (EMT) (**A**) Western blot results showed the expression levels of E-cadherin and Vimentin in NRK52e cells induced by 50 nM control mimics or miR542-3p; (**B**) Histogram showed the gray scale quantitative analysis for Western blot results; (**C**) Western blot results showed the expression levels of E-cadherin and Vimentin in NRK52e cells induced by 50 nM miR542-3p with or without 2.0 ng/mL TGFβ1; (**D**) Histogram showed the gray scale quantitative analysis for Western blot results. All data are displayed as means ± SD from three independent experiments, ** *p* < 0.01, *** *p* < 0.001.

TGFβ1 acts as a driving force for the kidney fibrosis [36,37,38,39] and was widely used for inducing a cell model of fibrosis or Epithelial-Mesenchymal Transition (EMT). Recently, investigations showed that the TGFβ1 signaling pathway could promote the maturation and expression of microRNAs [40,41]. To examine whether TGFβ1 can also influence the expression of miR542-3p *in vitro*, we investigated the miR542-3p expression in the NRK52e cell line pre-induced by different concentrations of TGFβ1 for 24 h (Figure 2A). We found that TGFβ1 activated the expression of miR542-3p. Real-time PCR results confirmed that TGFβ1 up-regulated miR542-3p expression in the NRK52e cell line and the optimal dose was 2 ng/mL (Figure 2A). Sequentially, we investigated the expression of the EMT marker and the fibrotic marker in the NRK52e cell line induced by different concentrates of TGFβ1. Interestingly, the expression tendency of Fibronectin (Figure 3D,F,G) and Vimentin (Figure 3E,F,G) were just like that of miR542-3p, while the expression tendency of BMP7 (Figure 3B,F,G) and E-cadherin (Figure 3C,F,G) in mRNA or protein levels showed to be obviously opposite to miR542-3p in the presence of TGFβ1.

### 2.3. MiR542-3p Induce Epithelial-to-Mesenchymal Transition (EMT)

To further investigate whether miR542-3p engaged in the progression of Epithelial-Mesenchymal Transition (EMT), we over-expressed miR542-3p and control mimics (Scramble mimics) in the NRK52e cell line respectively. We then examined the protein expression levels of the epithelial marker and mesenchymal marker by Western blot. As shown in Figure 3A, the epithelial marker E-cadherin was down-regulated, however the mesenchymal marker Vimentin was up-regulated in the NRK52e cell line induced by miR542-3p. Interestingly, the effect of miR542-3p may be enhanced by TGFβ1, which was shown in Figure 3C,D. Compared with that in NRK52e cells treated only with miR542-3p, the expression level of the epithelial marker reduced more significantly in cells treated with both miR542-3p and TGFβ1, but that of mesenchymal marker increased more apparently in cells treated with both miR542-3p and TGFβ1.

### 2.4. BMP7 Is a Direct Target of MiR542-3p

**Figure 4:**
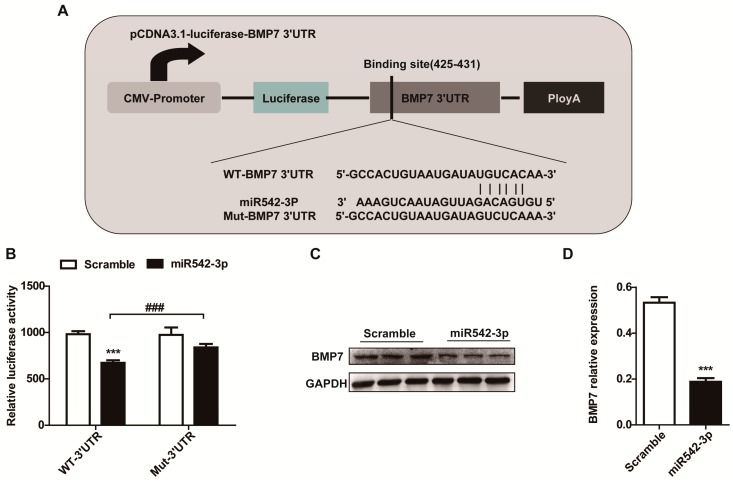
MiR542-3p targets BMP7 3′UTR and suppresses the expression of the BMP7 protein. (**A**) Schematic presentation of the miR542-3p binding site in the BMP7 3′UTR and wild type or mutant BMP7 3′UTR luciferase reporter vector structures; (**B**) Dual-luciferase reporter assays indicated that the luciferase activity was suppressed in 293T cells transiently transfected with miR542-3p and wild type BMP7 3′UTR reporter vector. However, the suppression of luciferase activity was eliminated when the binding site of miR542-3p was mutated in BMP7 3′UTR; (**C**) Western blot analysis of BMP7 expression levels in NRK52e cell transfected with control mimic or miR542-3p; (**D**) Histogram showed the gray scale quantitative analysis for Western blot results. All data are displayed as means ± SD from three independent experiments, **^###^** *p* < 0.001, *** *p* < 0.001.

In order to identify the potential regulatory targets of miR542-3p, several microRNA target prediction websites, including TargetScan, miRanda, and miRWalk, were conducted. Using these prediction websites, we identified BMP7 was a potential target gene of miR542-3p. The 3′UTR of BMP7 contained a putative target site for miR542-3p that is highly conserved among species (Figure 4A). To explore whether miR542-3p was directly targeting BMP7 3′UTR, we amplified the Human BMP7 3′UTR sequence with or without mutation of the miR542-3p binding-sites into pCDNA3.1-lucferase vector, which is a luciferase reporter vector. These reporters were transfected into a 293T cell along with miR542-3p or a negative control mimics (Scramble mimics) and we detected the luciferase activity by a Dual-Luciferase system. As expected, compared with the negative control, the relative luciferase activity had significantly decreased in the existence of miR542-3p and pCDNA3.1-luc-BMP7-3′UTR (Figure 4B). However, the suppression of luciferase activity was eliminated when the binding site of miR542-3p was mutated in BMP7 3′UTR (Figure 4B). To further confirm that miR542-3p was responsible for the decreasing of BMP7, NRK52e cells were pre-treated with miR542-3p or control mimic for 24 h. Western bolt assay showed that BMP7 expression dramatically reduced in NREK52e cells pre-induced by miR542-3p (Figure 4C,D). Further evidence of the impacts of miR542-3p on BMP7 was measured, and the BMP7 protein expression level was also down-regulated in the UUO mice model (Figure 1A) and the NRK52e cell line induced by TGFβ1 (Figure 2F). All results indicated that BMP7 is a target of miR542-3p.

### 2.5. Overexpression of BMP7 Attenuates the MiR542-3p-Induced EMT

**Figure 5:**
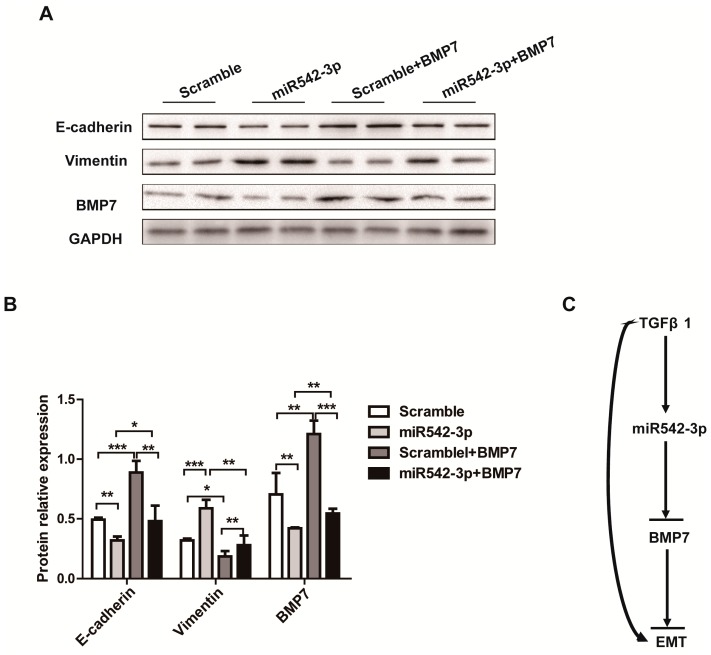
BMP7 attenuates the miR542-3p-induced EMT (**A**) Western blot results demonstrated that miR542-3p can suppress BMP7 protein expression and promote the Epithelial-Mesenchymal Transition (EMT) in the NRK52e cell line, however, the EMT was suppressed by the transiently transfected BMP7 expression vector (pCDNA3.1-BMP7); (**B**) Histogram showed the gray scale quantitative analysis for Western blot results. * *p* < 0.05, ** *p* < 0.01, *** *p* < 0.001; (**C**) The schematic diagrams of miR542-3p promoted Epithelial-Mesenchymal Transition (EMT).

Our next goal was to investigate whether miR542-3p promoted Epithelial-Mesenchymal Transition (EMT) via down-regulating the BMP7 protein expression through targeting BMP7 3′UTR. We then examined whether altering the BMP7 expression would affect the miR542-3p-induced Epithelial-Mesenchymal Transition (EMT) by transiently transfecting the BMP7 overexpression vector (pCDNA3.1-BMP7) into the NRK52e cell. As shown in Figure 5A,B, up-regulating miR542-3p in the NRK52e cell line can reduce the expression of E-cadherin and increase the expression of Vimentin, whereas elevating the BMP7 expression levels can rescue the E-cadherin expression and attenuate Vimentin expression (Figure 5A,B). These results implied that miR542-3p could promote Epithelial-Mesenchymal Transition (EMT) through targeting BMP7.

## 3. Discussion

The progress of chronic kidney diseases toward end-stage renal failure is one of the biggest social and clinical problems. In a progressive chronic renal diseases and kidney fibrosis mouse model (UUO), it was further validated that there was a potential common pathway contributing to tubulointeritial fibrosis and Epithelial-Mesenchymal Transition which included TGFβ signaling activation, ectopic microRNAs expression, and BMP7 signaling blockage [1,2,3,6,42,43,44,45]. Several researchers have reported that overexpression BMP7 protein [18,46,47] and several microRNAs [30,31] may be promising therapeutic methods for kidney fibrosis. However, the complicated molecular mechanisms of several microRNAs and BMP7 proteins determining the progression of EMT in kidney fibrosis remain largely unknown.

Accumulated evidence has linked miR542-3p to the complicated physiological process, such as cancer cell proliferation and apoptosis, suppression of osteoblast cell proliferation, and differentiation [20,21,22,23,26]. However, the knowledge on the precise relationship between miR542-3p and the process of Epithelial-Mesenchymal Transition in renal fibrosis were very limited. Recently, investigations indicated that miR542-3p can suppress cancer and inhibited cell proliferation through positively regulating p53 protein [22], a famous tumor suppressor gene, which plays a crucial role in governing many cellular events. Yemin Wang’s works told us that miRNAs, especially miR542-3p, may be another method of therapeutic cancer agent that can stimulate p53 expression and stabilization through disturbing the interactions between p53 and its negative regulator MDM2 [22]. As we all knew, p53 is a driving force of kidney fibrosis. As another reported potential target gene of miR542-3p is survivn [21,26] which suppresses apoptosis through an inhibited apoptosis protein by the caspase enzyme system [26]. Sena Yoon’s works indicated that miR542-3p played an important role as the regulator of the cell cycle through directly targeting survivin, and the cell cycle could be arrested at both the G1 and G2/M phases through the ectopic expression of miR542-3p [26].

In this paper, our results originally characterized that miR542-3p regulated Epithelial-Mesenchymal Transition (EMT) in NRK52e cells through down-regulating BMP7 protein expression by targeting BMP7 3′UTR. We firstly demonstrated that miR542-3p was increased in animal models of kidney fibrosis (Figure 1E) and its expression also could be activated by TGFβ1 *in vitro* (Figure 2A). In order to explain the role of miR542-3p in the kidney fibrosis and the progression of Epithelial-Mesenchymal Transition (EMT), we conducted an analysis of the expression of E-cadherin, a Vimentin protein in NRK52e cells, which pre-transfected miR542-3p or control mimics by Western blot assay. We found that the expression of the E-cadherin protein, an epithelial marker, was dramatically decreased. However, the expression of Vimentin, a mesenchymal marker, was increased in NRK52e cells, which was induced by miR542-3p (Figure 3A,B). To further clarify the molecular mechanism of miR542-3p in the progression of Epithelial-Mesenchymal Transition (EMT), we used several bioinformatics analysis websites to predict the target gene of miR542-3p. According to the results from the bioinformatics analysis, we found that BMP7 3’UTR contains a potential conserved binding site for miR542-3p in mammalians. To validate miR542-3p regulation of the progression of EMT by directing interaction with 3′UTR of BMP7, Dual-Luciferase report assay and Western blots were performed. Compared with control mimics and normal BMP7 3’UTR groups, the luciferase activity of miR542-3p and BMP7 3′UTR groups was dramatically reduced. However, when we mutated miR542-3p binding sites of BMP7 3′UTR, the luciferase activity by no means changed (Figure 4B). Lower expression of BMP7 protein levels were detected more in the NRK52E cells transfected with miR542-3p than in the control mimic (Figure 4C,D). Moreover, the progression of Epithelial-Mesenchymal Transition (EMT) induced by miR542-3p can be suppressed via a transiently transfected PCDNA3.2-BMP7 vector into the NRK52e cells (Figure 5A,B).

Our investigations demonstrated that the mechanism of miR542-3p regulating the EMT progression may be just as shown in Figure 5C. In kidney injury or disease, miR542-3p was up-regulated by the TGFβ1 signaling pathway or other stimuli, which then regulated the progression of EMT through interaction with BMP7 3′UTR. Moreover, this study characterized that the molecular mechanism of miR542-3p regulating EMT progression and demonstrated that inhibition of miR542-3p may be an effective therapy target of kidney fibrosis.

## 4. Experimental Section

### 4.1. Obstructive Kidney Disease Model

A UUO (unilateral ureteral obstruction) mouse model was performed by left ureteral ligation as described previously in eight week male C57BL/6J mice [36]. UUO or sham surgery was performed under 1% Pentobarbital anesthesia. The left ureter was exposed through a midline abdominal incision, then was tied off with fine suture at the mid-ureteral level to induce a complete obstruction.

All mice were obtained from Chongqing Medical University Animal House, China. The experimental procedures were approved in advance by the Chongqing Medical University Animal Ethic Committee.

### 4.2. Cell Culture and Transfection

The NRK52E, 293T cells were obtained from ATCC (Rockefeller, MA, USA). The NRK52E cell and HEK293T cells were maintained in DMEM/low glucose medium (Gibco, BRL Co., Ltd., Grand Island, NY, USA) supplemented with 1% penicillin/streptomycin (Life Technologies, Grand Island, NY, USA), 5% and 10% fetal bovine serum (*v*/*v*, GIBCO) respectively. All these cell lines were cultured at 37 °C in a humidified 5% CO_2_ atmosphere. The NRK52e cell lines were stimulated with or without human TGFβ1 (Sino Biological Inc., Beijing, China) at optimum concentration in a serum-free DMEM/low glucose.

The miR542-3p (UGUGACAGAUUGAUAACUGAAA), control mimics (scrambled miRNA control) and the miR542-3p primers were synthesized by GuangZhou RiboBio company in China. NRK52E cells were transfected with the miR542-3p mimics (50 nM) or control mimics (50 nM) with or without 2 µg pCDNA3.1-BMP7 via Lipofectamine 2000 (Invitrogen, Carlsbad, CA, USA) according to the manufacturer’s instructions before Western blots and real-time PCR analysis.

### 4.3. RNA Isolation and Quantitative Real-Time PCR Analysis

Total RNA was extracted using TRIzol reagent (Life Technologies, Grand Island, NY, USA) from kidney or cells. Further reverse transcription was performed by using a RevertAid First Srrand cDNA Synthesis kit (Thermo Scientific, Walham, MA, USA), according to the manufacturer’s instructions. The expression of miR542-3p was normalized to U6, and EMT and kidney fibrosis markers were calculated relative to 18sRNA. The mRNA levels of BMP7, E-cadherin, Fibronectin, Vimentin, and Collagen I were analysis by SYBR-Green qRT-PCR kit (TakaRa, Dalian, China) with the special primers. These RNA primers used as follows (Table 1).

**Table 1:** The sequences of the primers for real-time analysis.

Gene Name	Sense	Antisense
**BMP7**	CAGCCACCAGCAACCACT	GTCCATGCCGTCCAATCA
**Ecadherin**	GTCAACACCTACAACGCTGC	ACGTGCTTGGGTTGAAGACA
**Vimentin**	TGACCGCTTCGCCAACTA	CGCAACTCCCTCATCTCCT
**Collagen I**	AACTTTGCTTCCCAGATFTCCTATG	GCTTCCCCATCATCTCCATTCTTGC
**a-SMA**	GGGGTGATGGTGGGAATG	GGGGTGATGGTGGGAATG
**Fibnectin**	TGTGACCCAGACTTACGG	TGTAGGTGAACGGGAGAA
**U6**	CTCGCTTCGGCAGCACA	AACGCTTCACGAATTTGCGT
**18sRNA**	GTAACCCGTTGAACCCCATT	CCATCCAATCGGTAGTAGCG

### 4.4. Western Bolt Analysis

Kidney tissues and cell proteins were isolated with a RIPA lysis buffer (Beyotime, Shanghai, China) containing 1 mM PMSF. SDS-PAGE gel, and PVDF membrane (Millipore, Billerica, MA, USA) were prepared. Primary antibodies incubated separately with rabbit anti-E-cadherin (1:1000; Santa Cruz Biotechnology, Santa Cruz, CA, USA), BMP7 (1:1500; Proteintech, Wuhan, China), Vimentin (1:1000; Proteintech, Wuhan, China), GAPDH (1:2000; Proteintech, Wuhan, China). The membrane was incubated separately with anti-rabbit secondary antibody (1:2000, Zhong Shan Jin Qiao, Beijing, China) in a blocking solution after being washed with tri-buffered saline and tween 20 (TBST). Finally, we used the Western blot Chemiluminescent HRP Substrate (Immobilon Western, Billerica, MA, USA) to detect target proteins. Normalization of protein expression was used of internal control (GAPDH).

### 4.5. Dual Luciferase Reporter Assay

The full-length human BMP7 3′UTR was amplified by PCR using human genomic DNA extracted from the HKE293T cell as a template. The primers were sense primer CGACAGCTCTAATGTCATCC and anti-sense primer AACAGACTGAAGGAAGTCGG. We then constructed the mutant BMP7 3′UTR vector (pCDNA3.1-luc-BMP7 3′UTR-MUT) with sense primer AGAAGCCACTGTAATGATAGTCACAAATAAAACCCATGAAT and anti-sense primer TTTCATTCATGGGTTTTATTTGTGACTATCATTACAGTGGC. The 293T cell line was cultured in a 24-well cell culture cluster, and 50 nM miR542-3p or control mimics were co-transfected together with 0.5 µg pCDNA3.1-luc-BMP7 3′UTR or pCDNA3.1-luc-BMP7 3′UTR-MUT plasmid into cells per well via Lipofectamine 2000. Normalization was achieved with the co-transfection of 20 ng pRL-SV40 vectors per well. We measured the activities of relative luciferase with Dual-Luciferase Reporter Assay Systerm (Promega, Madison, WI, USA) according to the manufacturer’s instructions.

### 4.6. Statistical Analysis

The results were shown as means ± SD. Statistical significance was measured by performing an analysis of *t* test with statistical software Prism 5 (GraphPad, San Diego, CA, USA), and the values of *p* < 0.05 were considered as statistically significant.

## 5. Conclusions

In summary, in this study we have demonstrated that miR542-3p regulates EMT progression by directly targeting 3’UTR of BMP7. Moreover, our results suggest that miR542-3p may be a potential therapeutic target for anti-EMT.
